# Understanding dynamic friction through spontaneously evolving laboratory earthquakes

**DOI:** 10.1038/ncomms15991

**Published:** 2017-06-29

**Authors:** V. Rubino, A. J. Rosakis, N. Lapusta

**Affiliations:** 1 Graduate Aerospace Laboratories, California Institute of Technology, Pasadena, California 91125, USA; 2 Division of Engineering and Applied Science, California Institute of Technology, Pasadena, California 91125, USA; 3 Division of Geological and Planetary Sciences, California Institute of Technology, Pasadena, California 91125, USA

## Abstract

Friction plays a key role in how ruptures unzip faults in the Earth’s crust and release waves that cause destructive shaking. Yet dynamic friction evolution is one of the biggest uncertainties in earthquake science. Here we report on novel measurements of evolving local friction during spontaneously developing mini-earthquakes in the laboratory, enabled by our ultrahigh speed full-field imaging technique. The technique captures the evolution of displacements, velocities and stresses of dynamic ruptures, whose rupture speed range from sub-Rayleigh to supershear. The observed friction has complex evolution, featuring initial velocity strengthening followed by substantial velocity weakening. Our measurements are consistent with rate-and-state friction formulations supplemented with flash heating but not with widely used slip-weakening friction laws. This study develops a new approach for measuring local evolution of dynamic friction and has important implications for understanding earthquake hazard since laws governing frictional resistance of faults are vital ingredients in physically-based predictive models of the earthquake source.

## Abstract (web summary)

The role of friction is key to understanding how faults may rupture and generate earthquakes. Here, the authors capture the local evolution of dynamic friction in laboratory experiments via a new approach using a high-speed imaging technique, which may be used to understand the frictional resistance of faults.

Large destructive earthquakes are typically caused by dynamic rupture propagating along pre-existing faults in the Earth’s crust, such as the San Andreas Fault in California that appears to be ready for the next big earthquake^1^. During such rupture, the two sides of the fault slide past each other, accumulating relative displacement—or slip—with average slip velocities of the order of 1 m s^−1^. This marked process is heavily affected by dynamic friction and its dependence on rapidly evolving rupture parameters such as slip and slip rate. That is why dynamic friction is a key aspect of earthquake physics and a crucial input to models of both single dynamic ruptures and long-term earthquake behaviour^2^,^3^. Assumptions about dynamic friction can markedly change the interpretation of earthquake observations, leading to different conclusions about the physical mechanisms controlling rupture speeds, rupture modes, stress levels on faults, and patterns of seismic/aseismic slip^3^,^4^,^5^,^6^,^7^,^8^,^9^,^10^,^11^,^12^.

From theoretical and numerical studies, it is clear that friction needs to weaken for earthquake rupture to nucleate and proceed, but the detailed nature of appropriate friction laws are an active area of current study. One of the commonly used friction formulations is slip weakening^8^,^13^,^14^,^15^,^16^,^17^, in which friction decreases from a peak value to a residual value with increasing slip across the frictional interface. The formulation is a convenient and intuitive extension of the basic notions of static/kinetic friction and it is still actively used for numerical simulations, theoretical considerations and interpreting observations^8^,^13^,^14^,^15^,^16^,^17^. Higher-resolution experiments on propagating shear rupture^14^,^18^,^19^,^20^ show a more complex friction evolution with slip, with initial slip strengthening, followed by slip weakening. More elaborate slip-dependent friction models have been proposed to fit these details^14^,^18^,^19^,^20^. At the same time, a number of lab experiments have demonstrated significant dependence of friction on slip velocity, often mixed in with other effects^21^,^22^,^23^,^24^,^25^,^26^. Such friction experiments typically impose slip-velocity histories to the sample, assume uniform sliding along the interface, and measure the resulting averaged friction resistance. The expected friction evolution during earthquakes is determined from such measurements through formulations of empirical friction laws and subsequent dynamic rupture modeling.

Here we present and analyse our experimental measurements of local friction during spontaneously evolving dynamic rupture in a laboratory earthquake set-up. Although not spontaneously generated, our ruptures are spontaneously evolving, in the sense that their slip, slip rate, and shear stress evolution are not imposed but rather determined by the fault prestress, fault friction, and dynamic stress transfer during rupture. This is an important property of our experiments, since, typically, frictional studies are conducted by imposing the slip rate, slip, or stress histories. By capturing evolving friction as well as slip and slip rate during dynamic rupture of the laboratory fault, we can directly study the dependence of dynamic friction on slip and/or slip rate evolution characteristic of spontaneously evolving rupture. These measurements are enabled by our ultrahigh speed technique for imaging full-field spatial and temporal variations in displacements of the laboratory sample, from which we can infer full-field maps of stresses and particle velocities.

## Results

### Dynamic imaging of earthquakes in the laboratory

**Figure 1:**
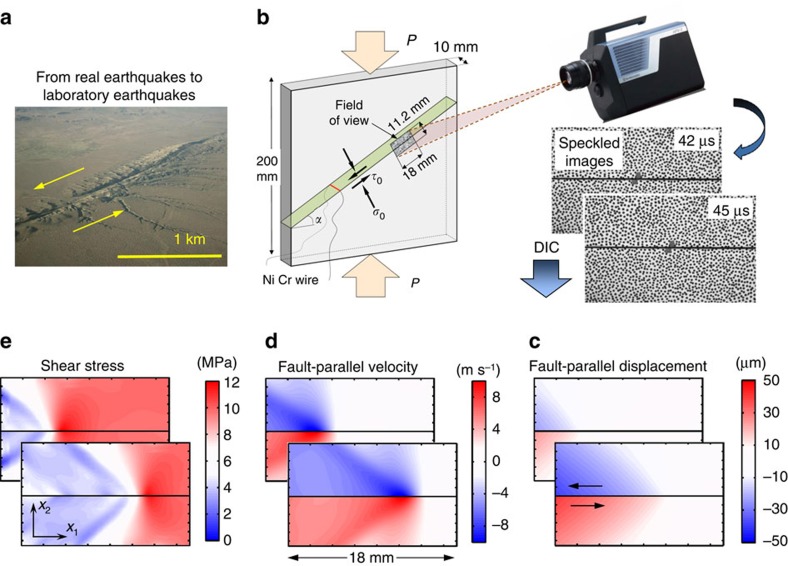
Imaging mini-earthquake ruptures with our ultrahigh speed full-field technique. (**a**,**b**) Earthquakes are mimicked in the laboratory by dynamic ruptures propagating along an inclined frictional interface, under the applied shear and normal prestresses simulating tectonic loading applied to a fault within the Earth’s crust. The level of prestress is controlled by the applied far-field loading *P* and interface inclination angle *α*. Part of the interface has a speckled pattern applied for the subsequent analysis. The picture of the San Andreas Fault, shown for visual comparison in **a**, is modified from www.sanandreasfault.org (Copyright (**c**) David K. Lynch). (**c**–**e**) The full-field time histories of displacements, velocities and stresses are experimentally obtained by capturing sequences of images with ultrahigh speed photography, and processing them with pattern-matching algorithms and highly tailored analysis. The case shown is for *P*=23 MPa and *α*=29°.

The spontaneously evolving dynamic ruptures are produced in the laboratory earthquake set-up that mimics the main features of a fault in the Earth’s crust^27^ (Fig. 1). The fault is simulated by an interface inclined at an angle *α* and prestressed both in shear and compression due to far-field load *P*, simulating tectonic stresses (as discussed further in the ‘Laboratory earthquake set-up’ section in Methods). Dynamic ruptures are nucleated due to the local pressure release provided by a rapid expansion of a NiCr wire filament due to an electrical discharge. To enable dynamic rupture initiation within a smaller, laboratory-scale sample, we use an analogue material, Homalite, which has a significantly (∼20 times) lower shear modulus compared to rocks. This is an important experimental advantage, since it significantly decreases all relevant critical length scales, such as the critical crack size and rupture nucleation size^16^, allowing us to study well-developed shear ruptures in samples of tens of centimeters, instead of several meters as would be required for rocks^28^. Previous versions of this laboratory earthquake set-up have been successfully employed to study a number of key dynamic rupture phenomena, including supershear transition, rupture directionality and limiting speeds due to bimaterial effect, off-fault attenuation and damage creation, and pulse-like to crack-like transition^9^,^15^,^29^,^30^,^31^.

**Figure 2:**
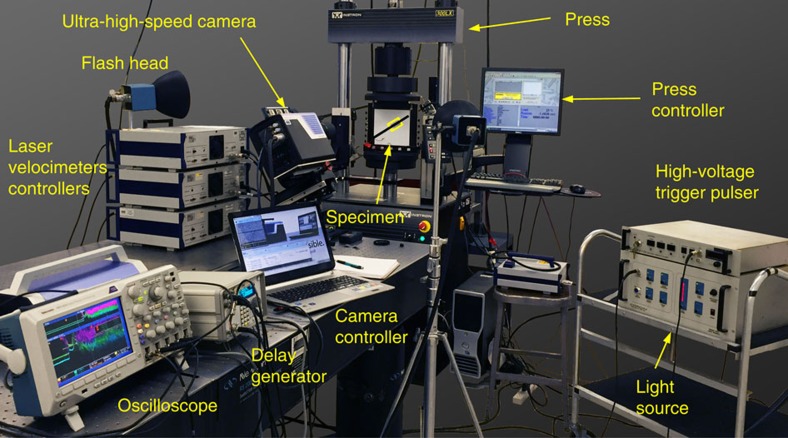
Laboratory earthquake set-up with the ultrahigh speed digital image correlation and laser velocimeter diagnostics. The sample contains an interface that mimics a crustal fault prestressed both in compression and in shear. Dynamic rupture is triggered through a local pressure release provided by a rapid expansion of a NiCr wire filament due to an electrical discharge. The Shimadzu HPV-X ultrahigh speed camera records images of the specimen during rupture propagation at 1–2 million frames per s. The well-developed technique of laser velocimetry is used for comparison of pointwise velocity measurements obtained with the full-field technique at selected locations.

The measurements presented here are enabled by our newly-developed technique for full-field imaging of dynamic ruptures (Figs 1 and 2). The specimen surface is coated by a carefully selected speckle pattern to produce a characteristic texture in the digital images. Digital images of the patterns distorted by the propagating rupture are acquired by a high-speed camera chosen for its lowest noise after an extensive comparison; 128 images are obtained with temporal sampling of 2 million frames per s. The sequence of images is turned into evolving displacement maps using the digital image correlation (DIC) method^32^ further developed for our experiments (see ‘Full-field imaging of dynamic ruptures’ section in Methods). The particle velocity and strain change maps are obtained through time and space differentiation of the displacement fields, respectively; the stress change maps are computed from the strain maps using known linear-elastic and high-strain-rate properties of the material tested^32^ (Fig. 1). To study friction evolution, we track the time history of the ratio of shear to normal stress along the fault (which gives the friction coefficient) together with those of slip and slip rate across the interface. The total stresses are computed by adding the (non-uniform) stress changes inferred from the imaging and (uniform) prestress values computed from the imposed far-field load *P* and inclination angle *α* (see section ‘Post-processing of the displacement fields’ in Methods). The uniform distribution of prestresses in our experiments has been verified in earlier studies directly using photoelasticity and indirectly through repeatability of ruptures in different experiments performed under the same far-field experimental loading^29^,^33^. The uniformity of prestress is further supported by the near-steady rupture propagation through our observation window as discussed in the following.

**Figure 3:**
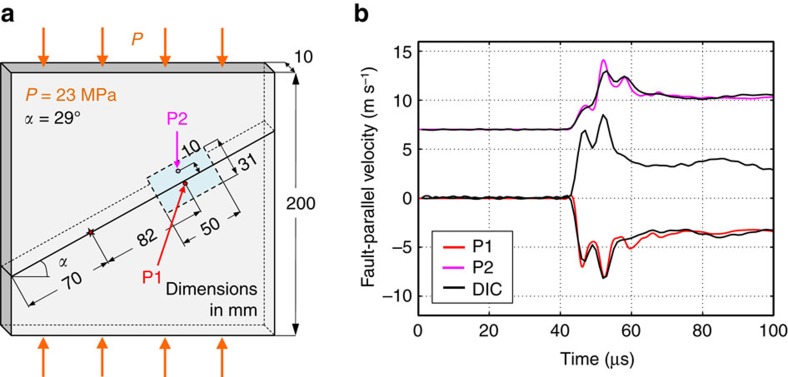
Validation of digital image correlation method with laser velocimeters. (**a**) Schematics of the configuration employed for the comparison of the full-field imaging method to the laser velocimeter technique. The field of view (blue rectangle, 50 × 31 mm^2^) and the location of the velocimeter measurements (P1 and P2) are indicated. (**b**) Fault-parallel velocity time-histories measured with laser velocimeters (colored curves) and with the full-field DIC technique (black curves). The curve corresponding to the velocimeter location P2 is shifted upwards by 7 m s^−1^ for clarity. Note the excellent agreement of the two measurements. The two black curves based on the full-field technique from above and below the interface show a near-perfect anti-symmetric signal, consistent with in-plane shear rupture.

The full-field images reveal the details of rupture propagation within the entire deformation window with a spatial and temporal resolution that was previously obtainable only in numerical simulations (Fig. 1; Supplementary Movie 1). By following the rupture tip travelling along the interface, we can determine the rupture speed. For example, in the experiment presented in Fig. 1, we find that the rupture is supershear^15^, as evident by the Mach cone features observed in the shear stress maps. We have verified the accuracy of the full-field DIC measurements by comparing the velocity time-histories at selected locations (Fig. 3) with simultaneous, independent point-wise measurements using the well-developed technique of laser velocimetry^30^,^34^. The DIC and laser-velocimeter measurements are in excellent agreement (Fig. 3b), a remarkable development since the laser velocimeters are specifically designed to accurately resolve the rapid variations in particle velocities^30^,^34^. However, because of the cost and laser arrangements involved, it is only feasible to employ 2–3 velocimeters in each experiment. Now, the validated DIC technique provides us with tens of thousands of time-history measurements arranged into a two-dimensional spatial picture of particle velocities, deformations, and stresses, enabling further insight into a range of dynamic earthquake phenomena that can be studied with this experimental set-up. For example, the full-field data can be used to study how rapidly the motion attenuates away from the fault for different rupture scenarios, an important aspect of seismic hazard assessment.

### Evolution of dynamic friction with slip

**Figure 4:**
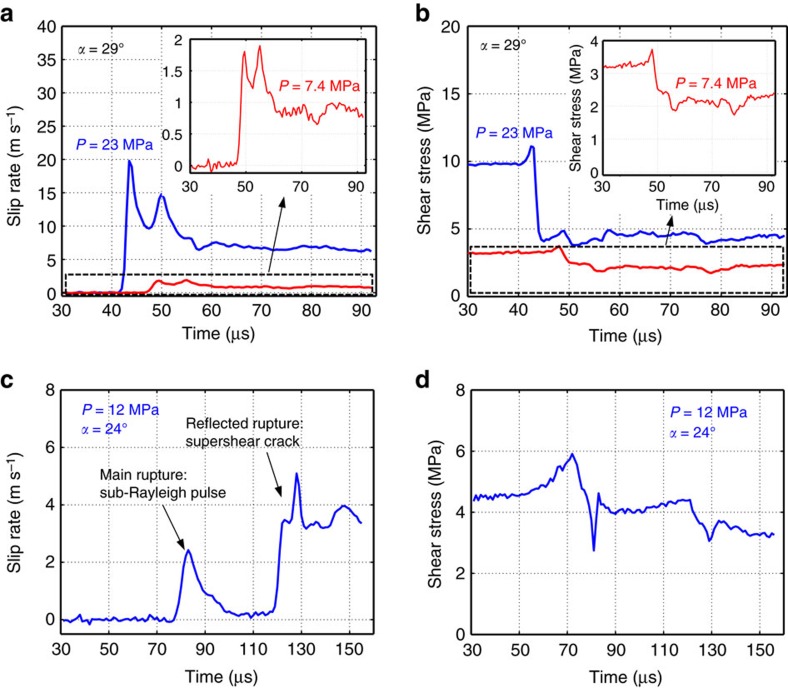
Monitoring the laboratory earthquake vitals. (**a**) Slip rate and (**b**) shear stress time histories obtained on the interface at the center of the field of view shown in Fig. 1 for two far field loads, *P*=23 MPa (blue) and *P*=7.4 MPa (red), and angle *α*=29°; both cases result in supershear ruptures. Insets: Magnified slip rate and shear stress time histories for the case *P*=7.4 MPa. The rupture produced by the higher far-field load has an order of magnitude higher peak slip rate and a more prominent reduction in shear stress compared to the lower load case. Yet the two ruptures are qualitatively similar as demonstrated by the insets. (**c**) Slip rate and (**d**) shear stress time histories for *P*=12 MPa and *α*=24°. This experiment has a very different slip-rate history, with a sub-Rayleigh pulse-like rupture propagating first, followed by a reflected supershear crack-like rupture.

**Figure 5:**
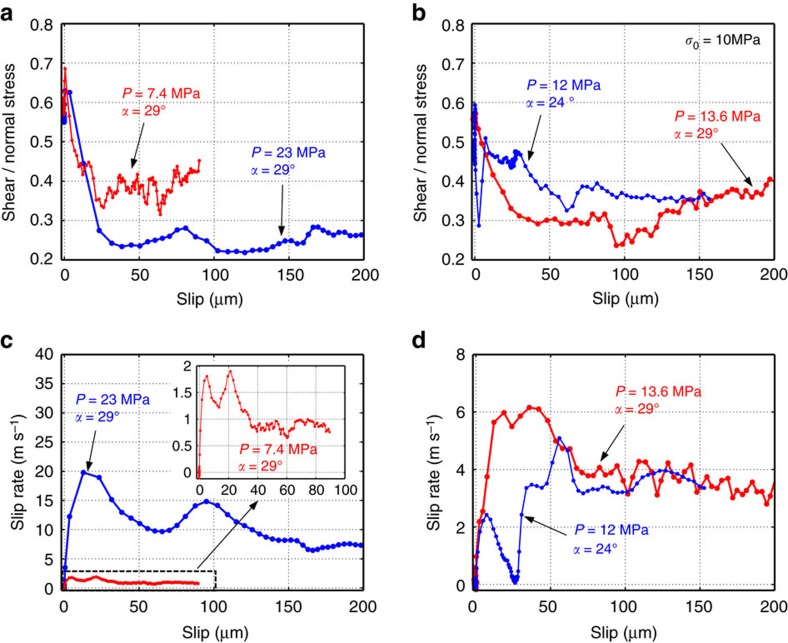
Rupture evolution with slip. (**a**,**b**) Evolution of friction (= shear to normal stress ratio during slip) with slip on the interface at the center of the field of view shown in Fig. 1. The four experimental ruptures have different dependence of friction on slip, indicating that the friction cannot be described by a purely slip-dependent law. (**c**,**d**) Evolution of slip rate versus slip for the same ruptures. The four ruptures are characterized by significantly different slip-rate histories, which result in different friction at any given value of slip.

Here we focus on the evolution of dynamic friction, and specifically whether it is controlled by slip or slip rate. Our experimental set-up enables us to produce spontaneously evolving dynamic ruptures with significantly different slip-rate histories, which result in remarkably different friction behaviour (Figs 4 and 5). We start by analysing two tests conducted under two different far-field loading conditions (*P*=7.4 and 23 MPa) but the same inclination angle *α*=29°; both cases result in supershear ruptures. For the two loads, rupture exhibits a rapid increase of slip rates towards a peak value and subsequent decay to near-constant values (Figs 4a and 5c), although the level of slip rates is quite different between these two cases: the peak slip rate is ∼2 and ∼20 m s^−1^ for the lower and higher load case, respectively. The rupture produced by the higher far-field load also has a higher overall level of shear stress and a more pronounced reduction in shear stress compared to the lower load case (Fig. 4b). Note that in both cases, as the rupture front approaches, the shear stress initially increases with slip rate, from its static pre-stress level, and subsequently drops to a lower dynamic level.

The dependence of friction on slip in the two experiments described above is displayed in (Fig. 5a). For each of these experiments, this dependence qualitatively resembles that described by a linear slip-weakening friction law. However, the variation of friction with slip is drastically different in the two cases, demonstrating that a unique, purely slip-dependent law cannot describe the frictional characteristics of the interface. Indeed, all of our experiments, when reviewed collectively, do not support slip weakening as the operant law in friction.

To illustrate this further, let us consider another experiment, with the far-field loading *P*=12 MPa and inclination angle *α*=24°, which features a sub-Rayleigh pulse-like rupture followed by a supershear crack-like rupture, as evidenced by the slip-rate history (Fig. 4c). In this experiment, once nucleated, the rupture propagates bilaterally along the interface, with two rupture tips traveling in opposite directions. The rupture traveling towards the higher edge of the specimen, and through our imaging region, propagates as a pulse at sub-Rayleigh speeds. The timing analysis shows that the following supershear crack-like rupture is the result of the other rupture tip reflecting from the specimen lateral surface and transitioning to supershear speeds; it then enters our imaging region. As in the previous examples, the shear stress initially increases from its static prestress level, as the rupture front approaches, and then drops and eventually settles to a lower dynamic value (Fig. 4d). When the supershear crack-like rupture arrives, the interface slips at a higher rate and the shear stress evolves again in a similar pattern: it first increases and then drops. Hence, in this experiment, the friction evolution with slip exhibits two weakening episodes, associated with the passing of the two ruptures—the sub-Rayleigh pulse and the supershear crack (Fig. 5b,d). Again, a purely slip-weakening law is not capable of reproducing such complex friction behaviour.

### Evolution of dynamic friction with slip rate

**Figure 6:**
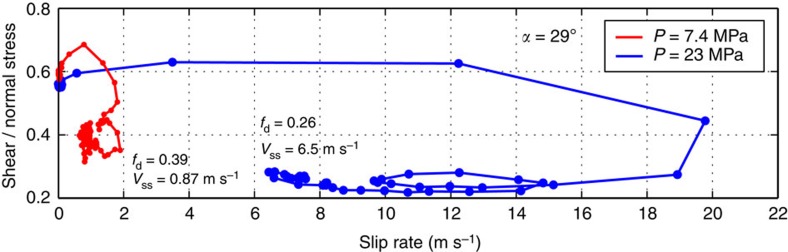
Evolution of dynamic friction with slip rate. Friction evolution is presented for the experiments at two far-field loads, *P*=7.4 MPa (red) and *P*=23 MPa (blue) and angle *α*=29°. These curves correspond to the two ruptures shown in Figs 4a,b and 5a,c. Both cases show initial strengthening with slip rate (direct effect) followed by rate weakening, as captured by rate-and-state formulations. Note that the steady-state level of dynamic friction depends on the slip rate (*f*_d_=0.39 for *V*_ss_=0.87 m s^−1^ and *f*_d_=0.26 for *V*_ss_=6.5 m s^−1^).

To further understand the features of observed friction, let us consider its variation with slip velocity (Fig. 6). Clearly, the friction evolution cannot be described by a purely rate-dependent law either; in fact, each case displays its own distinct hysteretic behaviour indicating strong sliding history dependence. Despite their differences, the two cases display qualitatively similar behaviour characterized by: (i) initial strengthening with the slip velocity, consistent with the direct effect of the rate-and-state friction formulations^21^, (ii) subsequent strong weakening with slip velocity, and (iii) near constant level of dynamic friction that depends on the sustained value of the slip rates (*f*_d_∼0.39 for *V*∼0.87 m s^−1^ and *f*_d_∼0.26 for *V*∼6.5 m s^−1^). The second peak in the slip-rate function (Fig. 4a), which is likely due to the finite thickness of the specimen^30^, results in a secondary ‘loop’ in the friction versus slip rate curves, with some strengthening (consistent with the direct effect of rate-and-state friction) followed by weakening. Note that the peak friction coefficient is not reached at incipient slip, as assumed in many slip-dependent friction formulations, but rather after slip has initiated on the interface.

Overall, this behaviour is qualitatively consistent with the rate-and-state friction laws, in which friction is the function of the slip rate and a state variable that describes the evolution of contact population^21^ (see section ‘On friction laws’ in Methods). In these laws, friction is rate-dependent after sufficient slip at a constant slip rate, but exhibits history-dependent transient effects during changes of velocity that are mathematically represented by the evolving state variable. During dynamic rupture, interfaces governed by the rate-and-state friction laws exhibit friction evolution similar to what occurs in our experiments, with the friction first increasing due to the direct effect, then decreasing due to evolution of the state variable, and then remaining constant for constant dynamic slip velocities due to friction being velocity-dependent in steady state^35^.

Some slip-weakening formulations linked higher values of the effective weakening slip distance to higher values of normal stress^36^. Our measurements indicate that the effective slip weakening depends on the slip-rate history rather than on the level of normal stress. In the experiment with a pulse-like rupture followed by a crack-like rupture, the two ruptures display a notably different effective weakening with slip (Fig. 5b), despite occurring at the same normal stress. The same conclusion is reached by comparing two tests conducted under different levels of far-field loading *P* and different inclination angle *α* but having the same level of resolved normal stress on the interface. The two tests are characterized by substantially different slip rate histories (Fig. 5d). The resulting friction versus slip evolution is substantially different, and the effective slip scale over which friction decreases is substantially different in the two experiments (Fig. 5b), despite the same normal stress, further substantiating the point that the slip-rate evolution is the dominating factor controlling the evolution of friction with slip.

### Near-steady rupture propagation through observation window

**Figure 7:**
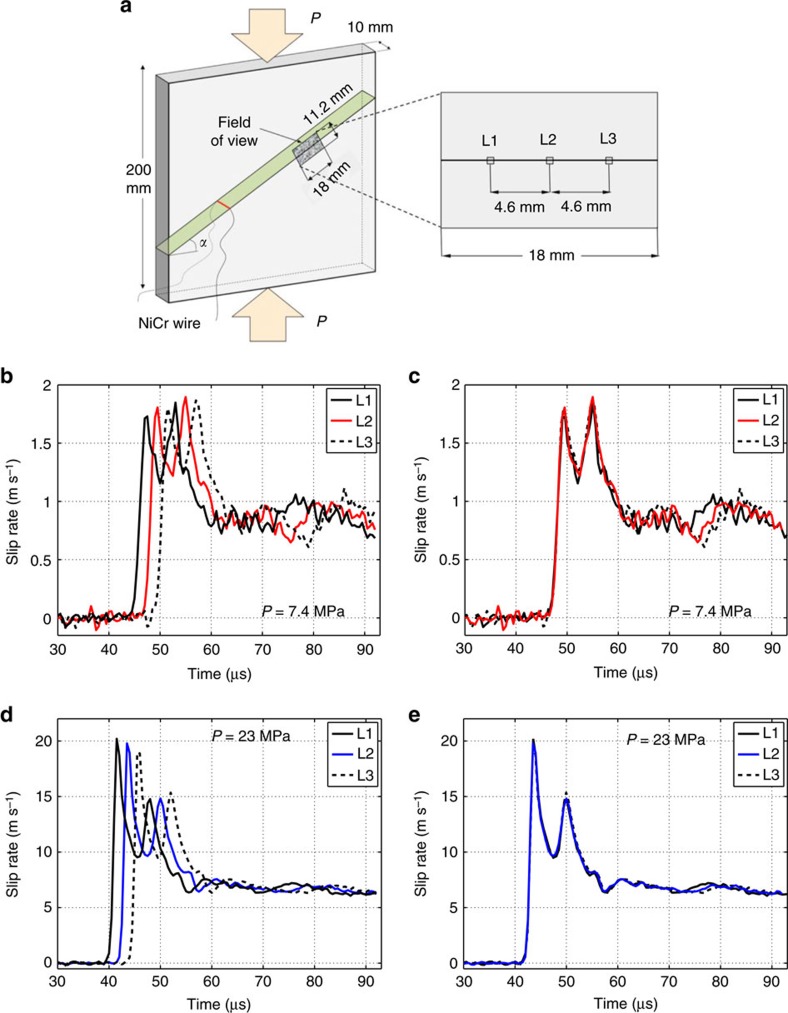
Steady propagation of spontaneously evolving ruptures. (**a**) Sketch of the specimen configuration showing the field of view and the locations corresponding to the slip-rate curves. (**b**–**d**) Slip-rate time histories (averaged over 1 mm) obtained at the three selected locations along the interface, for *P*=7.4 MPa (**b**) and *P*=23 MPa (**d**) respectively. (**c**–**e**) The time histories at locations L1 and L3 are time shifted (respectively, forward and backward) by Δ*t*=*d*/*V*_r_, where *d* is the distance between L1 and L2 (and also L2 and L3) and *V*_r_ is the rupture speed. The near-perfect overlap of the shifted time histories at locations L1 and L3 with the time history at L2 shows that the ruptures are well developed and propagate in a steady fashion through our field of view.

Our measurements show that the slip-rate and friction time histories considered are quite similar for different points along our observation window, indicating that the ruptures are steady and well-developed. To illustrate the similarity, we compare the time histories at the center of the field of view with two other locations along the interface, at a distance *d*=4.6 mm from the center of the imaged area (Fig. 7) and find that the time histories are nearly identical. Hence the rupture is well-developed and steady when it enters our field of view, and our friction analysis does not depend on the location along the observation window. The fact that slip rate time histories at different locations along the interface are nearly identical supports a uniform distribution of normal and shear prestress along the interface, as a heterogeneous state of prestress would cause variations in the slip-rate time histories.

### Enhanced dynamic weakening

**Figure 8:**
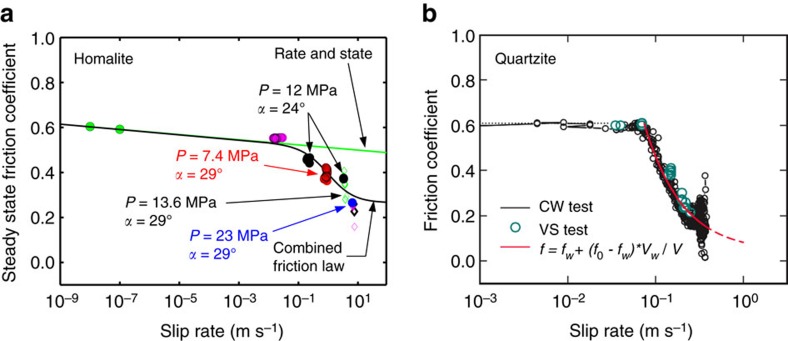
Steady-state friction versus slip rate. (**a**) Experimental measurements of steady-state friction coefficient versus slip rate and fits with the standard rate-and-state friction formulation (green curve), and combined formulation of rate-and-state friction enhanced by flash heating (black curve). Our steady-state measurements are consistent with the combined formulation. Green dots are low-velocity measurements obtained in collaboration with Kilgore, Beeler, and Lu and reported in Lu^39^. Red, blue, black and purple solid symbols are measurements obtained with the smallest field of view used in this study (18 × 11.2 mm^2^) and hence the highest level of accuracy. The green, black and purple empty diamonds are measurements with larger fields of view (up to 145 × 91 mm^2^) and lower levels of accuracy. (**b**) Experimental measurements of dynamic friction on quartzite samples^22^, showing similar behaviour for rocks, with significant slip-rate dependence of friction for high steady-state slip rates, consistent with the flash heating formulation. Note the different horizontal scale for the two plots.

Values of friction achieved in our experiments at different constant slip rates reveal pronounced weakening with slip rate (Fig. 8a) suggesting activation of enhanced dynamic weakening in our experiments. For example, at the higher sustained slip rate (*V*∼6.5 m s^−1^), the friction coefficient decreases from a peak value of ∼0.63 down to ∼0.26, variation that cannot be explained with standard, logarithmic rate-and-state formulations that generally result in mild friction changes^21^ (see sections ‘Steady-state friction analysis’ and ‘On friction laws’ in Methods).

We find that our results are consistent with a combined formulation of rate-and-state friction enhanced with flash heating weakening^2^,^22^,^37^,^38^ (Fig. 8a). Flash heating is a dynamic mechanism in which tips of the contacting asperities heat up and dramatically weaken due to shear, resulting in a pronounced, 1/*V*, dependence of friction in slip velocity *V*:


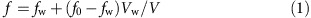


where *V*_w_ and *f*_w_ are the weakening slip velocity and the residual friction coefficient, respectively, and *f*_0_ is the friction coefficient for *V*<*V*_w_. In the combined formulation of rate-and-state friction enhanced with flash heating weakening, the low-velocity friction coefficient *f*_0_ of equation (1) is described by rate-and-state friction (see section ‘On friction laws’ in Methods). In fitting the experimental results with the rate-and-state and combined formulations, we use the rate-and-state properties constrained for Homalite interfaces by low-velocity friction experiments^39^ (Fig. 8a).

Hence our measurements clearly indicate that friction weakens with slip rate much more prominently than predicted by logarithmic rate-and-state friction laws. While our data are consistent with the combined formulation of rate-and-state friction enhanced by flash weakening, we are not able to determine whether the actual physical mechanism operating is indeed flash heating, and there may be other enhanced velocity-weakening mechanisms at play. Note that the systematic rate dependence shown in Fig. 8a contains points from both sub-Rayleigh and supershear ruptures, which have quite different elastodynamic stress fields, but propagate over the interface with the same preparation and hence the same friction properties.

It is remarkable that the inferred enhanced velocity-weakening parameters are consistent with the previous experimental study of Lu *et al*.^29^ that constrained the steady-state rate-weakening properties of our experimental interfaces based on how the rupture changes from crack-like to pulse-like as the nondimensional interface prestress, in terms of the ratio of the resolved normal to shear stress, is reduced. The origin of the pulse-like mode of rupture propagation in natural earthquakes is a key issue in earthquake physics^6^,^9^. Enhanced velocity weakening due to flash heating has been predicted to produce pulse-like ruptures on interfaces with low prestress^6^,^9^, suggesting that such weakening can both explain the origin of slip pulses and resolve the heat paradox^6^,^9^,^11^,^22^,^37^. Our direct measurements of the evolving friction confirm the conclusions of Lu *et al*.^29^ that the pulse-like ruptures observed in their experiments for lower nondimensional prestresses are due to substantial velocity weakening of friction. Indeed, our experiments feature a main pulse-like rupture for the lower-prestress case of *α*=24°.

To summarize, our experimental measurement demonstrate, for the first time, that friction evolution with slip velocity is consistent with the combined rate-and-state and flash-heating weakening formulation based on measurements performed locally during a spontaneously evolving rupture, rather than from a combination of classical friction experiments where different sliding velocities are imposed from the testing apparatus and assumed to be uniform over the slipping surface. Our measurements on Homalite, a polymer, also suggest the generality of the flash-heating formulation, which was initially proposed in engineering tribology to interpret dry friction in metals^40^, and then studied in earthquake science as a candidate mechanism contributing to friction evolution during seismic slip^6^,^11^,^22^,^37^. Indeed, there is a remarkable qualitative similarity between our measurements obtained on a polymer and those obtained on quartzite rock^22^ (Fig. 8a,b).

### On interpreting our experiments with slip-dependent laws

The two experiments presented in Fig. 5a display friction evolution with slip resembling linear slip-weakening friction (equation (11) in Methods). According to linear slip weakening, the dynamic friction coefficient *f*_d_ and the characteristic slip distance *D*_c_ are material parameters and, as a consequence, the dependence of friction on slip is the same for a given interface. Instead, our measurements show that the dependence of friction on slip is different for different slip-rate histories (Fig. 5), indicating that friction cannot be described by a purely slip-dependent law. In the two experiments of Fig. 5a, which appear closest to the linear slip weakening, the apparent dynamic friction coefficient *f*_d_ and slip weakening distance *D*_c_ are different, indicating that they are not material properties, but rather effective quantities that depend on the dynamics of the process.

Our measurements indicate that the slip scale over which friction decreases depends on the slip-rate history but not directly on the level of normal stress, in contrast with what inferred by other authors^36^. If such slip scale depended on the normal stress, two measurements performed under the same level of normal prestress *σ*_*0*_, but different slip-rate histories, would exhibit the same slip scale. Our tests show that this is not the case, as illustrated by the following two examples. In the case with a pulse-like rupture followed by the supershear crack-like rupture (*P*=12 MPa and *α*=24°, Figs 4c and 5b,d), the slip over which weakening occurs is significantly different between the initial pulse-like and the following crack-like parts of the rupture, despite the normal stress being the same. Another example is provided by a test with an applied far-field loading *P*=13.6 MPa and inclination angle *α*=29°, which has the same value of the resolved normal stress on the interface, *σ*_0_=*P* cos^2^ *α*=10 MPa, as the test at *P*=12 MPa and *α*=24°. While these two tests are conducted under the same level of interface-normal stress, they have substantially different slip rate histories (Fig. 5d), evolution of friction with slip (Fig. 5b), and slip scale over which friction decreases (Fig. 5b).

We conclude that both the slip-weakening length scale and the (variable) dynamic level of friction depend on the slip-rate history, and hence the linear slip-weakening friction law is not an adequate description of friction evolution.

### On interpreting our experiments with flash heating

The weakening velocity *V*_w_ in the flash heating process for our Homalite interface can be estimated as^37^:


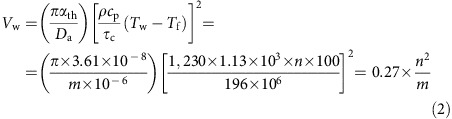


where *α*_th_=3.61 × 10^−18^ m^2^ s^−1^ (ref. 39) is thermal diffusivity, *ρc*_p_=1.39 × 10^6^ J m^−3^ K^−1^ (ref. 39) is heat capacity per unit volume, *D*_a_=*m*μm is the average contact diameter (with *m*=1–10), *τ*_c_=0.1*μ*=196 MPa is the shear strength of the contact (estimated as one tenth of the shear modulus of the bulk^41^), and *T*_w_−*T*_f_=*n* × 10^2^ K is the temperature change that activates flash heating, which could be several hundred degrees for Homalite.

Given that *m*=1–10 and *n*=1–3, the weakening velocity *V*_*w*_ is estimated to be of the order of 0.1 to 1 m s^−1^. Note that this estimate is uncertain, since even the assumed values of the thermal diffusivity, heat capacity and shear contact strength may be different for the specific Homalite used in our study.

For the combined formulation of rate-and-state friction supplemented with flash heating (equation (18)), the least-squares fit of our steady-state friction measurements (Fig. 8a) leads to 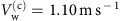
and 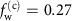. This fitted value of the characteristic weakening velocity is broadly consistent with the estimate above for reasonable *n* and *m* values, for example, *n*=2 and *m*=1. The thermal properties of Homalite need to be known in greater detail to provide a more constrained comparison that could indirectly substantiate the flash heating explanation for weakening.

It is not possible to observe flash heating directly in our experiments due to a number of experimental and technological limitations. Flash heating happens when tips of asperities (which are of the order of 1 μm) quickly heat up during contact and then cool down when not in contact. This highly transient heating over micrometer scale cannot be detected with available experimental diagnostics and leaves no post-mortem signature. That is why we conclude that the experimental measurements are consistent with the flash-heating formulation, but we cannot conclusively claim that flash heating actually occurs in our experiments.

## Discussion

Our findings conclusively demonstrate that, during spontaneously evolving dynamic rupture, friction has complex evolution with substantial velocity weakening at high, seismic slip rates. Consequently, purely slip-dependent friction formulations cannot capture the evolution of dynamic friction. At the same time, friction is not purely rate-dependent. At the high slip rates tested in our study, standard, logarithmic rate-and-state friction models can predict the initial strengthening behaviour but not the substantial weakening that follows. The steady-state friction behaviour at high slip rates can be captured with the formulation developed for enhanced weakening due to flash heating, which appears to be valid for a wide range of materials, including metals^40^, rocks^22^, and, as shown in this work, polymers. This points towards universality of dynamic friction and makes our measurements relevant to many engineering and materials science applications involving friction, such as composite materials failure by fibre pull-out.

Our results have important implications for earthquake physics, validating approaches in which experimental results for simplified slip and slip-velocity histories are combined to study the overall dynamic rupture behaviour. Furthermore, the substantial weakening observed at seismic slip rates in the laboratory is likely to operate during natural earthquakes and could explain the lack of heat flow observed on some active faults, such as the San Andreas Fault. At the same time, friction on natural faults can be affected by a number of additional factors that are not accounted for in our present laboratory set-up, including the presence of fault gouge, pore fluids and off-fault damage^2^. The novel experimental approach to dynamic friction measurements developed in this work can be used to study the effects of some of these factors on dynamic friction, by introducing damage in the bulk^42^, adding rock gouge to the specimen interface, and inducing multiple ruptures in the same specimen.

## Methods

### Laboratory earthquake set-up

The laboratory earthquake set-up mimics a fault in the Earth’s crust loaded in compression and shear by the frictionally held interface of two Homalite quadrilateral plates. A square plate of Homalite-100, with the dimensions 200 mm × 200 mm × 10 mm, is cut using computer-numerical-control (CNC) milling, producing an interface of inclination angle *α* (Fig. 1). The mating surfaces of the interface are subsequently polished to a near-optical grade finish, in order to erase any manufacturing marks coming from the computer-numerical-control cutting. The surfaces are then roughened by employing a micro-bead blasting procedure with abrasive glass media having diameters in the range of 104–211 μm (refs 30, 32). This protocol ensures consistent surface roughness and repeatability of the dynamic frictional rupture experiments. New test specimens are used in every test. The two Homalite quadrilateral plates are brought into contact and compressed with a uniaxial load *P* (Fig. 1). The applied loading *P*, in conjunction with the inclination angle *α*, control the level of shear *τ*_0_=*P* sin *α* cos *α* and normal *σ*_0_=*P* cos^2^ *α* prestress on the fault. The non-dimensional prestress is given by *τ*_0_/*σ*_0_=tan *α*. Nucleation of dynamic rupture is obtained through a local pressure release provided by a rapid expansion of a NiCr wire filament due to an electrical discharge.

To provide a characteristic texture for image matching, the specimen’s surface is first coated with a uniform layer of white paint and then decorated with a random black-speckle pattern. To resolve sharp displacement gradients, a small speckle size is required. On the other hand, too small speckles would result in aliasing. This results in an average desired speckle size of 3–6 pixels^43^. Since we image areas of different dimensions, the speckle size is adapted to each case to be consistently in the range of 3–6 pixels. An example of speckled images is provided in Fig. 1.

The high-speed diagnostics consists of an ultrahigh-speed camera system, capable of up to 10 million frames per s, a high-voltage pulse generator to discharge the NiCr wire and initiate the rupture, and a high-speed white light source system (Fig. 2). A sequence of 128 digital images of the specimen during rupture propagation is acquired using a Shimadzu HPV-X camera, at 1 to 2 million frames per s, depending on the experiment, and with a resolution of 250 × 400 pixels. The images discussed in this work are taken over an area ranging from a minimum of 18 × 11.2 mm^2^, which are typically recorded at 2 million frames per s, up to 145 × 91 mm^2^, recorded at 1 million frame per s. In addition, for selected experiments, laser heterodyne interferometers are employed to accurately measure particle velocities at up to two locations in one experiment^30^,^34^.

### Full-field imaging of dynamic ruptures

In order to produce a full-field characterization of the dynamic ruptures, we employ the digital image correlation method (DIC). The DIC method is an optical technique, which analyses digital images by tracking, with sub-pixel accuracy, the motion and deformations of image windows containing a characteristic grey-level signature^43^. We use the correlation software VIC-2D (Correlation Solutions Inc.) enhanced with the ‘Fill-Boundary’ algorithm to treat interface discontinuities. The correlation analysis is performed by comparing the specimen’s image, taken before rupture, to each subsequent deformed image. The displacement fields are then computed with respect to the chosen reference configuration. Two key parameters in performing the correlation analysis are the subset size and step size. Pattern matching is performed over image subsets to regularize the non-uniqueness of the pixel-by-pixel correlation problem. The subset size is the size of the image window whose motion and deformation is tracked by the correlation algorithm. For each subset, the solution provides the two in-plane displacement components at the subset center. Smaller subset sizes result in finer spatial resolution, while too small subsets do not contain enough gray-level information and result in larger errors. The subset size choice also depends on the signal-to-noise ratio (SNR); tests with larger SNR can afford smaller subset sizes. The step size is the distance between the centers of two nearest subsets. Smaller step sizes increase the density of correlation results. For example, the two tests presented in Fig. 4a,b were analysed with a subset size of 41 pixels (1.9 mm) and 51 pixels (2.3 mm) for the case of larger (*P*=23 MPa) and smaller applied loading (*P*=7.4 MPa) respectively. The step size was 1 pixel (46 μm) for both cases.

The images are correlated over two independent rectangular domains, separated at the specimen interface. This is because employing one domain containing the interface would imply using subsets across the interface, averaging displacements on the opposite sides of the interface and preventing us from capturing the discontinuities across the interface. Since the correlation solution is found for the subset center, the standard VIC-2D algorithm would only be able to produce the displacement map up to half a subset away from the interface. The ‘Fill-Boundary’ algorithm, developed by Correlated Solutions Inc. with our input, uses affine transform functions to extrapolate the displacements all the way to the interface.

### Post-processing of the displacement fields

Displacement fields are filtered with the non-local-means (NL-means) filter^32^,^44^,^45^. In contrast with local filters, which smooth each pixel with neighboring pixels regardless of their content and are not capable of capturing discontinuities, the NL-means filter accounts for the ‘context’ around each pixel. This is achieved by considering windows (neighbourhoods) around each pixel and comparing them to neighboring windows. The windows are then averaged with Gaussian weights, where larger weights are assigned to windows that express a higher degree of similarity. This procedure enables efficient image denoising, preserving sharp features and large gradients. The NL-means filter operates with the following input parameters: the size of the neighborhood *N*, the search area dimension *Ω*, which defines the span over which the search of similar neighbourhoods is computed, and the noise parameter *h*, related to noise level of the signal. In all cases analysed in this study, we use: *N*=3 × 3 pixels, Ω=21 × 21 pixels, and *h*=0.5. We found that a second iteration of the NL-means filter with the same parameters helped to further smooth the displacement fields without lost of information. We have also investigated and checked the effect of filtering parameters both on the displacement and strain fields. The above reported parameters achieve displacement smoothing yet maintaining intact the original signal pattern. An example of the sequence of two images is shown in Fig. 1.

Strains are computed from the filtered displacement fields using the finite difference approximation. Away from the boundaries, we use the central difference scheme:


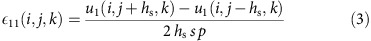



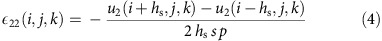



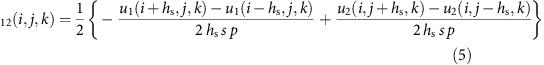


where *u*_1_(*i*, *j*, *k*) and *u*_2_(*i*, *j*, *k*) are the fault-parallel and fault-normal displacement components, respectively (Fig. 1c shows the fault-parallel component), for pixel (*i*, *j*) and frame *k*, expressed in μm; 2*h*_s_ defines the stencil size and *s* is the step size, both expressed in pixels; *p* is the pixel size, which for the two cases presented in the main text is 46 μm. Here we take *h*_s_=1 pixel. Close to the interface, we use the backward or forward difference scheme to compute strains above and below the interface, respectively. Below the interface, the forward difference approximation reads:


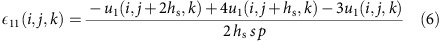



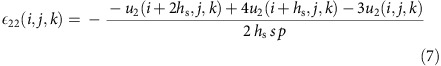



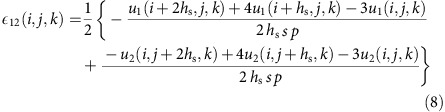


Stress fields are computed from strain fields using the standard plane-stress linear elastic constitutive equations (Fig. 1e shows the shear stress component). Since Homalite is a viscoelastic material, we use the dynamic Young’s modulus *E*_d_=5.3 GPa (ref. 46) to compute the dynamic stress change^47^,^48^, together with a Poisson’s ratio of *ν*=0.35 (refs 32, 49). Since the displacement fields are computed using the loaded specimen configuration as reference, the strains and stresses computed from these fields are changes over the reference configuration. To recover the actual level of stress, we add the computed resolved normal and shear stress, in the reference configuration, to the DIC measured stresses as:


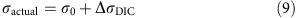



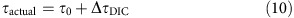


This procedure is justified by the fact that the resolved levels of shear and normal stress are nearly uniform along the interface, as discussed in the main text.

The particle velocity maps (Fig. 1d) are obtained through time differentiation of the displacement fields (Fig. 1c), using the central difference scheme. The slip and slip rate values are computed by subtracting the displacement and particle velocity values, respectively, at the pixels immediately above and below the interface. The friction coefficient is computed by the shear to normal stress ratio at the pixels along the interface.

### Averaging procedure

The slip, slip rate, traction components and friction values are initially computed for all pixels along the interface. To produce the time histories and friction evolution curves presented here, we average the slip, slip rate, and stress components over a 1 mm region at the center of the imaged area, which is at a distance of 82 mm from the rupture nucleation location. This procedure reduces potential numerical oscillations of the correlation solution. The averaging of the time histories is achieved by time shifting each curve by Δ*t*=(*x*_l_−*x*_c_)/*V*_r_, where *V*_r_ is the rupture speed, *x*_l_ denotes a generic location and *x*_c_ denotes the center of the imaged area. The time histories corresponding to locations around the center of the imaged areas are collapsed over the time history corresponding to the center of the field of view, and the curves thus obtained are subsequently averaged. For the tests discussed in the main text, the averaging over 1 mm involves 22 curves. This procedure is performed for all time histories of interest and it is used to produce the time histories of Fig. 4, the friction versus slip curves of Fig. 5a,b, and the friction versus slip rate curves of Fig. 6. Averaging time histories over 2 and 4 mm regions produces the same results as those obtained with 1 mm averaging, showing both that the signals are already well smoothed and that the rupture is well developed, propagating in a steady fashion. Note that the comparison of slip rate histories at different locations presented in Fig. 7 is performed on curves averaged over a 1 mm region, according to the procedure outlined above.

### Steady-state friction analysis

In this section, we detail the procedure used to determine the steady-state friction coefficients and steady-state slip rate data points of Fig. 8a. To find steady state values, we select windows of sustained near-constant slip rate, with the slip rate variation, Δ*V*, satisfying 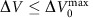 over the entire window. 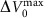 is the maximum variation in slip rate measured before rupture arrival; since that slip rate should be physically zero, 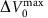 gives an estimate of the measurement error. For the near-constant slip rate to be sustained, the window should contain a minimum number of data points *n*_dp_. The minimum number of data points guarantees that the steady state is maintained over a minimum time interval before slip rate starts evolving again. We use *n*_dp_=10. Note that our time histories are made of 128 data points, corresponding to the recorded camera frames.

The steady state results presented in Fig. 8a comprise four tests performed with the same field of view: 18 mm × 11.2 mm (solid symbols in Fig. 8a). We also performed additional experiments with larger fields of view. While these experiments provide more insight, they are characterized by a lower signal to noise ratio. We perform the same steady-state analysis for them as for the other tests and include the corresponding data points in the steady-state friction versus slip rate plot (empty symbols in Fig. 8a). These steady-state measurements have a larger scatter, yet they follow the same trend as determined with the smaller and more accurate set of data points.

### On friction laws

One of the most common formulations of friction is that of slip weakening. The slip-weakening formulation has been introduced by analogy to cohesive-zone models of mode I cracks as well as based on experimental results^50^,^51^,^52^,^53^,^54^. A common simple form of the law, the so-called linear slip weakening, prescribes a linear variation of the friction coefficient *f* from the static value *f*_s_ according to:


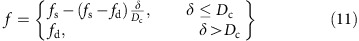


where *f*_d_ is the dynamic friction coefficient, and *D*_c_ is the slip over which *f*_d_ is reached. In this formulation, the parameters *f*_d_ and *D*_c_ are material parameters. For a detailed discussion on these parameters, see the section ‘On interpreting our experiments with slip weakening’.

In the widely used rate-and-state friction laws, developed for relatively slow slip rates compared to the seismic range, friction depends on the slip rate *V* and evolving state variable that represents memory effects^21^,^55^,^56^,^57^,^58^,^59^,^60^:


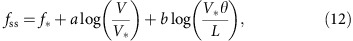



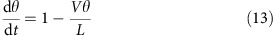


where f_*_ is the friction coefficient at the reference velocity V_*_, *a* and *b* are rate and state parameters, and *L* is the characteristic slip for the state variable evolution. Several evolution laws for the state variable have been proposed, including the aging law^55^,^56^,^57^, given above, the slip law^57^ and the composite law^61^,^62^. In part, rate-and-state friction incorporates a direct strengthening effect in response to rapid slip rate increases, which can potentially explain the initial strengthening in our experiments (Fig. 5a,b). Note that this formulation results in the dependence of friction on slip similar to slip weakening in the case of slip-rate histories characteristic of the rupture front (Fig. 5a)^35^,^63^. At steady state, the rate-and-state law takes the form:


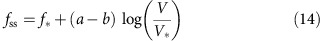


We plot this expression in Fig. 8a to compare it with our steady state measurements, using f_*_=0.58, V_*_=1 μm s^−1^, (*a*−*b*)=−0.005 reported by Lu^39^. The measurements were obtained for smaller samples with the same interface preparation procedure in velocity-jump experiments.

Experiments on rocks show that seismic rates (*V*>0.1 m s^−1^) are characterized by enhanced rate weakening, dramatically reducing the friction coefficient. One weakening mechanisms with extensive theoretical and experimental support is flash heating^2^,^22^,^37^,^38^. According to the flash heating friction law, the friction coefficient evolves as:


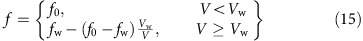


with


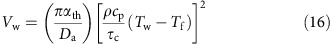


where *V*_w_ is the characteristic weakening velocity, *f*_0_ is the friction coefficient for *V*<*V*_w_, *f*_w_ is the residual friction coefficient, *α*_th_ is the thermal diffusivity, *D*_a_ is the average contact diameter, *ρ* is the density, *c*_p_ is the heat capacity, *τ*_c_ is the shear strength of individual contacts, *T*_w_ is the characteristic weakening (e.g., melting) temperature, and *T* is the average temperature of the slip surface. Note that the theoretical estimate of *V*_w_=0.1 m s^−1^ for rocks^37^ matches the experimentally inferred one. Note also that the estimate does not depend on the normal stress. The normal stress dependence of *V*_w_ has been observed in experiments^25^ but in the context of melt-welt formation.

One can combine the rate-and-state expressions at the low slip rates and flash heating at the high slip rates by assuming that *f*_0_ of the flash heating formulation is given by the rate-and-state formulation and replacing *V* in the flash heating formulations with *L*/*θ*, an expression that evolves towards *V* with slip^64^,^65^. The combined formulation reads:


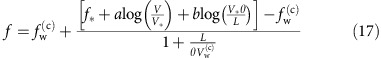


At steady state, the combined friction law takes the form:


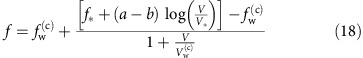


where 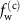
and 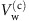 are, respectively, the residual friction coefficient and the weakening slip velocity for the combined formulation.

### Data availability

All relevant data supporting the findings of this study are available from the corresponding author upon reasonable request.

## Additional information

**How to cite this article:** Rubino, V. *et al*. Understanding dynamic friction through spontaneously evolving laboratory earthquakes. *Nat. Commun.* **8,** 15991 doi: 10.1038/ncomms15991 (2017).

**Publisher’s note:** Springer Nature remains neutral with regard to jurisdictional claims in published maps and institutional affiliations.

## Supplementary Material

Supplementary Information
