# Publisher Correction: Understanding dynamic friction through spontaneously evolving laboratory earthquakes

**DOI:** 10.1038/s41467-019-08566-7

**Published:** 2019-02-06

**Authors:** V. Rubino, A. J. Rosakis, N. Lapusta

**Affiliations:** 1 Graduate Aerospace Laboratories, California Institute of Technology, Pasadena, California 91125 USA; 2 Division of Engineering and Applied Science, California Institute of Technology, Pasadena, California 91125 USA; 3 Division of Geological and Planetary Sciences, California Institute of Technology, Pasadena, California 91125 USA

Correction to: *Nature Communications;* 10.1038/ncomms15991; published online 29 Jun 2017.

The HTML version of this Article incorrectly omits Supplementary Movie 1. Supplementary Movie 1 can be found as Supplementary Information associated with this Correction.

## Supplementary information

Supplementary Movie 1

