# Supplementary material for: Understanding dynamic friction through spontaneously evolving laboratory earthquakes
Source: Nat Commun. 2017 Jun 29;8:15991. doi: 10.1038/ncomms15991 (PMC5493769; doi:10.1038/ncomms15991)
Supplement: Supplementary Information [file ncomms15991-s1.pdf]

File name: Supplementary Movie 1
Description: Fault-parallel velocity
